# Relation of peritubular capillary features to class of lupus nephritis

**DOI:** 10.1186/s12882-016-0388-2

**Published:** 2016-11-09

**Authors:** Sirirat Anutrakulchai, Tanin Titipungul, Thanyaluk Pattay, Putachart Mesung, Anucha Puapairoj, Dhavee Sirivongs, Cholatip Pongsakul, Prasit Futrakul, Bandit Thinkhamrop, Richard J. Johnson

**Affiliations:** 1 Division of Nephrology, Department of Internal Medicine, Faculty of Medicine, Khon Kaen University, Khon Kaen, Thailand; 2 Department of Pathology, Mahasarakham Hospital, Maha Sarakham, Thailand; 3 Mission Hospital Phuket, Phuket, Thailand; 4 Department of Pathology, Faculty of Medicine, Khon Kaen University, Khon Kaen, Thailand; 5 Renal Microvascular Research Group, King Chulalongkorn Memorial Hospital, Chulalongkorn University, Pathum Wan, Bangkok Thailand; 6 Data Management and Statistical Analysis Center (DAMASAC), Epidemiology and Biostatistics, Faculty of Public Health, Khon Kaen University, Khon Kaen, Thailand; 7 Division of Renal Diseases and Hypertension, University of Colorado, Denver, USA

**Keywords:** Peritubular capillary, Lupus nephritis

## Abstract

**Background:**

Experimental studies have linked peritubular capillary (PTC) loss with progression of chronic kidney disease. Minimal information on PTC in lupus nephritis (LN) has been reported. We therefore evaluated the PTC area in different classes of LN and determined if specific clinical characteristics correlated with PTC changes.

**Methods:**

Renal biopsies of 253 subjects with LN (categorized using the ISN/RPS 2003 classification) and 13 normal renal donors (the controls) were retrospectively evaluated for PTC morphology by staining for CD31 with immunohistochemistry method. The percent positive area of PTC (% PTC) was correlated with serum and urinary measures of renal function and renal pathology.

**Results:**

Significant PTC loss was observed in all classes of LN compared to controls. The % PTC area was highest in controls (7.64±1.48 %) with levels of 1.95±1.50, 4.16±3.85, 4.19±4.45, 5.02±1.79, and 4.45±3.75 in classes II, III, IV, IV combined with V and V, respectively (all *p* values < 0.05). The lowest PTC density was observed in class II LN, but this may be because some cases with worse classes of LN showed increased PTC density due to abnormally dilated capillaries associated with acute inflammation and angiogenesis. %PTC was increased in those with hematuria (5.8±5.2 vs. 3.6±3.4 %, red blood cells 3-10 vs. < 3 cells/high power field, *p* < 0.05) and was reduced in those with a moderately declined renal function (3.29±3.40 vs. 4.42±4.12, eGFR 15-59 vs. ≥ 60 ml/min/1.73 m2, *p* < 0.05). Nephrotic-range proteinuria also trended to be associated with lower PTC density although it did not reach statistical significance (3.1±2.6 vs. 4.9±4.5, *p*= 0.067).

**Conclusions:**

LN is associated with PTC loss and the severity correlates with reduced renal function. Further studies are needed to investigate whether a loss of PTC can predict long term renal outcomes in LN.

**Electronic supplementary material:**

The online version of this article (doi:10.1186/s12882-016-0388-2) contains supplementary material, which is available to authorized users.

## Background

Lupus nephritis (LN) can present with diverse clinical manifestations from asymptomatic proteinuria or hematuria to nephrotic and nephritic syndromes. Immune mediated renal injury can lead to local inflammation followed by progressive scarring from loss of intrinsic renal parenchymal cells [1, 2]. Renal injury in LN is known to affect glomerular, tubulointerstitial, and vascular compartments [2, 3]. In 2003, the International Society of Nephrology/Renal Pathology Society (ISN/RPS) classified LN into 6 classes according to the respective glomerular pathological characteristics [4, 5]. In addition to the predominantly glomerular involvement in LN, renal vascular lesions may affect glomerular capillaries, arterioles and arteries leading to thrombotic microangiopathy, lupus vasculopathy and lupus vasculitis [3, 6–9], while tubulointerstitial injury manifests as interstitial inflammation, renal tubular atrophy and/or interstitial fibrosis [10, 11]. Several studies suggest that the severity of vascular and tubulointerstitial injury in LN predicts renal outcome [3, 6–12]. However, information on the integrity of the peritubular capillaries (PTC), which is the primary vascular supply for the renal tubules, has been limited to date.

Progressive PTC loss causes tubulointerstitial hypoxia which induces the processes of inflammation, tubular cell apoptosis and finally tubulointerstitial fibrosis [13]. Decreased PTC accompanied by declining renal function has been reported in various human kidney diseases (e.g., diabetic glomerulosclerosis, mesangioproliferative glomerulonephritides, amyloidosis, benign nephrosclerosis and chronic interstitial nephritis) [14]. With renal injury one may also observe transient increases in PTC due to angiogenesis associated with acute inflammation, as has been reported in kidneys associated with urinary tract obstruction and reflux nephropathy and in mice with reduced renal mass [15, 16].

Thacker et al. reported a decreased density of glomerular capillaries and PTC associated with down-regulation of intrarenal vascular endothelial growth factor-A (VEGF-A) in 20 renal biopsies of class II-V LN patients [17]. Hayagawa et al. also reported a pattern of peritubular capillaritis in a SLE patient presenting with tubulointerstitial nephritis [18]. We therefore evaluated a large cohort (>200 biopsies) of LN to determine the nature of the PTC and whether there may be an association with clinical characteristics or pathological classes of LN. Our hypothesis was that PTC loss would be associated with reduced renal function.

## Methods

### Study design

This was a cross-sectional retrospective study reported according to the STROBE (STrengthening the Reporting of OBservational studies in Epidemiology) guidelines.

### Patients

The study included all of the patients ≥15 years of age with renal pathologies diagnosed as LN at Srinagarind Hospital, Khon Kaen, Thailand, between January 1, 2008 and December 31, 2011. The medical records and laboratory data collected during the two- week pre-biopsy period were reviewed for clinical variables (including estimated glomerular filtration rate (eGFR), 24 h-urine protein and urinalysis). The eGFR was calculated using the Chronic Kidney Disease Epidemiology Collaboration formula [19].

### Renal histopathology

The renal biopsies were classified according to the histological types of LN using the ISN/RPS 2003 classification. LN class VI was excluded from the study because there was only one specimen. The classes of LN and severity of tubulointerstital damage were determined by the pathologist (AP) who was blinded to the study. The extent of tubular atrophy in the renal cortex and the areas of interstitial inflammatory cells infiltration and fibrosis were scored according to degree of involvement: 0 (none); 1 (≤25 %); 2 (26–50 %), and 3 (>50 %). Thirteen renal biopsies obtained during implantation of kidneys from deceased kidney donors showing normal renal histology were used as the normal controls for PTC count.

### Renal immunohistochemistry

Immunoperoxidase staining using anti-CD31 antibody (to represent PTC) was performed on renal biopsies of LN patients and the normal renal donors (the controls). CD31, also known as PECAM-1 (platelet endothelial cell adhesion molecule 1), is a 130-kDa transmembrane glycoprotein and an endothelial marker. Previous studies in patients with primary glomerulonephritis identified differences in CD31 expression in glomerular capillaries and PTC in various glomerular diseases [20–22]. Additionally, Izmirly et al. performed immunoperoxidase of anti-CD31 for evaluation the density of PTCs in the renal cortex of LN patients [23].

### Method of immunohistochemistry

Sections were deparafinized, rehydrated in a graded series of ethanol, soaked in 3 % hydrogen-peroxide in methanol for 5 min to block endogenous enzyme activity and washed for 5 min with phosphate buffered saline (PBS). Sections were subjected to antigen retrieval by immersing them in 10 mM citrate buffer (pH 6), followed by microwave treatment at heat level 10 (the highest) for 3 min and level 3 (the mean) for 10 min. After completing the cycles, the sections were allowed to cool at room temperature (~25 °C) for 20 min then rinsed in running tap water, distilled water, and PBS. After pre-treatment, the sections were treated with PBS containing 3 % normal horse serum at room temperature for 20 min, then incubated with mouse anti- human CD31 protein dilution 1:100 (Neomarker, Labvision Corporation, USA), for 1 h at room temperature. After washing with PBS, the sections were incubated with peroxidase-conjugated Envision™ antibody (DAKO, Glostrup, Denmark) for 30 min and the color developed with 0.1 % diaminobenzidine tetrahydrochloride (DAB) solution. The sections were then lightly counterstained with Mayer’s Haematoxylin, rinsed in water for 3–4 min, dehydrated, cleared and mounted in Permount®.

### CD31 quantification

Slides were scanned at 20 × magnification using a slide scanner instrument (Scanscope XT S/N 1469, Aperio). The scanned images were saved in 24-bit color TIFF format. The whole scan images were viewed at high resolution using the Aperio System’s annotation software (ImageScope 10, Aperio). Two pathologists (TT and PM) used the ImageScope drawing tools to assess the PTC positive area in a blinded fashion. The protocol of software (Positive Pixel Count, Aperio v9) was configured to detect the number of pixels that show weak, moderate and strong threshold limits in the brown colorimetric channel. Glomeruli, large vessels and fibrous tissues were excluded from the selection. The percent positive area of PTC (%PTC) was calculated using the following equation: the total number of positive pixels for CD31 at PTC divided by the total number positive and negative of pixels multiplied by 100. The inter-rater agreement of the two pathologists in quantification of %PTC on 40 renal samples showed significant concordance with a correlation coefficient of 0.96.

### Statistical analyses

Statistical analyses comprised computing (a) the frequency counts and percentages for the categorical variables and (b) the means and standard deviations for the continuous variables. The two-tailed *t* test and ANOVA were used to compare the continuous variables of the two groups and multiple groups, respectively. Categorical data were compared by the chi-squared and Fisher’s exact test. Multivariate linear regression analysis was performed by stepwise backward elimination to determine the clinical factors significantly associated with changes in PTC. *P*-values < 0.05 were considered statistically significant. All statistical analyses were performed using SPSS for Windows version 17.0 and STATA version 14.0.

## Results

A total of 253 LN patients were included (83.8 % female). The overall mean age was 33.7 ± 11.7 years (median 33 years with range 15 to 68 years). The histological types and associated demographics are presented in Table 1. According to the ISN/RPS 2003 LN classification, there was a respective 23, 6, 171, 3, and 50 cases in class II, III, IV, IV combined with V (IV + V), and V. The mean SBP was highest in the class IV group (140 ± 24 mmHg), whereas the respective level in the class II, III, IV + V and V groups was 129 ± 15, 134 ± 22, 125 ± 19 and 125 ± 16 mmHg. Subjects with LN class IV group had a lower mean eGFR than the other groups (i.e., 59 ± 38 mL/min/1.73 m^2^). The 24-h urine protein of patients in the class II (2.6 ± 1.5 g/d) and III (1.9 ± 0.7 g/d) were lower than the class IV (3.7 ± 2.9 g/d) and V (3.5 ± 2.1 g/d) groups. Microscopic hematuria was more frequent in class III and IV LN patients. Tubular atrophy, interstitial inflammatory cell infiltration and interstitial fibrosis were highest in class IV LN patients (Table 1).

**Table 1:** Clinical data at the time of renal biopsy of lupus nephritis patients classified according to the ISN/RPS 2003. Data expressed as mean ± SD unless otherwise specified

Lupus nephritis classes (*N* = 253)	Class II (*N* = 23)	Class III (*N* = 6)	Class IV (*N* = 171)	Class IV + V (*N* = 3)	Class V (*N* = 50)
Age at biopsy (years)	34 ± 10	39 ± 14	33 ± 12	35 ± 10	36 ± 12
Male : Female (n)	3:20	2:4	27:144	0:3	9:41
SBP (mmHg)	129 ± 15	134 ± 22	140 ± 24^b^	125 ± 19	125 ± 16^h^
DBP (mmHg)	80 ± 9	86 ± 17	86 ± 16^b^	82 ± 16	80 ± 14
Blood urea nitrogen (mg/dL)	13.9 ± 6.9	33.0 ± 21.3^a^	41.9 ± 32.4^b^	21.4 ± 3.2^c,g^	19.4 ± 13.8^d,f,h^
Serum creatinine (mg/dL)	0.75 ± 0.21	1.60 ± 1.17^a^	1.92 ± 1.49^b^	0.83 ± 0.15^g^	0.80 ± 0.33^f,h^
eGFR (mL/min/1.73 m^2^)	109 ± 22	71 ± 44^a^	59 ± 38^b^	95 ± 25	102 ± 30^f,h^
Serum albumin (g/dL)	3.27 ± 0.54	3.20 ± 0.23	2.65 ± 0.81^b,e^	2.87 ± 0.81	2.81 ± 0.88
Serum complement (mg/dL)					
C3 concentration	102 ± 47	108 ± 45	67 ± 102^b^	57 ± 28	97 ± 45^h^
C4 concentration	25 ± 18	26 ± 15	16 ± 18	8 ± 4^c,g^	22 ± 13^h,i^
Urinary protein (g/24 h)	2.60 ± 1.51	1.85 ± 0.70	3.69 ± 2.85^b,e^	3.23 ± 1.70	3.53 ± 2.05^d,f^
Urinary RBC (cells/HPF)	19 ± 39	45 ± 62	26 ± 35	4 ± 3^g^	4 ± 7^d,f,h^
Urinary WBC (cells/HPF)	10 ± 26	17 ± 30	13 ± 24	5 ± 8	3 ± 5^f,h^
Tubular atrophy scores (% of patients)^&&^ 0/1/2	67/27/6	33/67/0	25/40/35	33/33/33	47/38/15
Interstitial inflammatory cells infiltration scores (% of patients)^&&^ 0/1/2/3	71/29/0/0	33/33/17/17	28/58/12/2	33/33/33/0	77/23/0/0
Interstitial fibrosis scores (% of patients)^&&^ 0/1/2/3	67/27/13/0	33/67/0/0	28/66/4/2	33/33/33/0	55/32/13/0

Notes: Estimated glomerular filtration rate calculated using the CKD-EPI creatinine 2009 formula. SBP; systolic blood pressure, DBP; diastolic blood pressure, eGFR; estimated glomerular filtration rate, RBC; red blood cell, WBC; white blood cell, HPF; high power field. *p* < 0.05 ^a^class II vs. III, ^b^II vs. IV, ^c^II vs. IV + V, ^d^II vs. V, ^e^III vs. IV, ^f^III vs. V, ^g^IV vs. IV + V, ^h^IV vs. V, ^i^IV + V vs. V. ^&&^all *p* < 0.01 by Fisher’s exact test

**Fig. 1:**
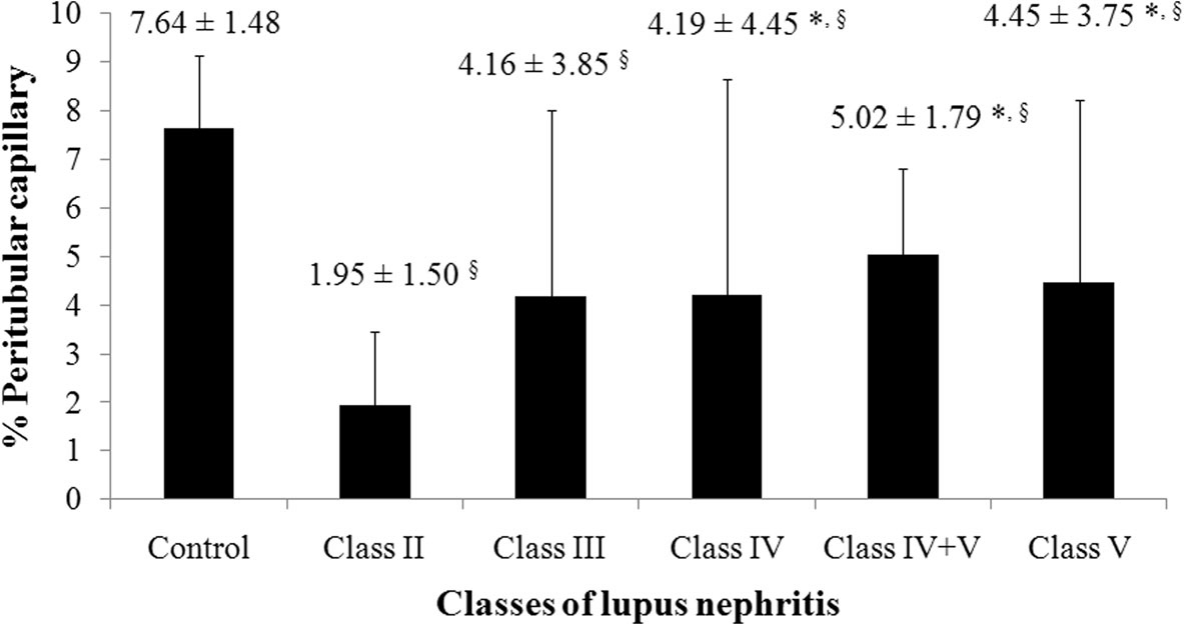
Percentage of peritubular capillaries in controls vs. various classes of LN. Notes: *p < 0.05 vs. class II, ^§^ *p* < 0.01 vs. controls

**Fig. 2:**
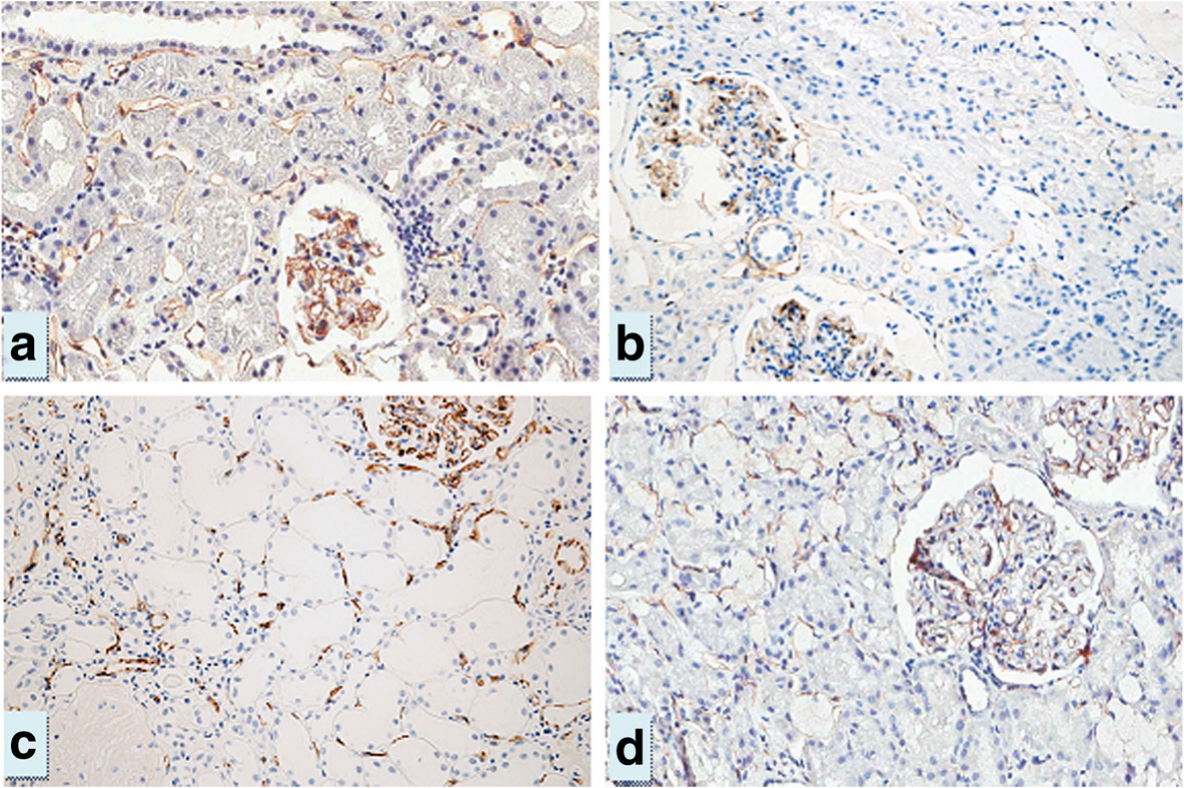
CD31 expression in the peritubular capillaries (PTC) demonstrated in the control (**a**, %PTC = 7.6), class II LN patient (**b**, %PTC =2.0), class IV LN patient (**c**, %PTC = 4.2) and class V LN patient (**d**, %PTC =4.5). Magnification 200×

**Fig. 3:**
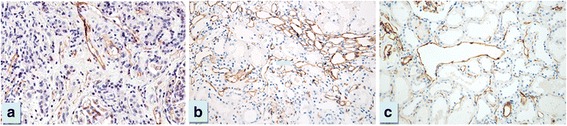
The control revealed normal % PTC (**a**), some cases of class IV LN (**b**) and class V LN (**c**) demonstrated high %PTC with abnormal morphology of dilated PTC. (Magnification 200×)

**Fig. 4:**
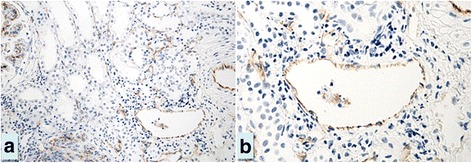
Figures **a** and **b**—the magnification figure of **a**—represented the dilated PTC and some demonstrated mononuclear cell infiltration within and around the PTC, the characteristic of peritubular capillaritis. (Magnification 200× for Figure **a** and 400× for Figure **b**)

The mean age of thirteen normal controls was comparable with LN patients (31.4 ± 17.7 years) and the range for PTC density (%PTC) was 6.02 - 9.90 % with the mean of 7.64 ± 1.48 %. The %PTC of all LN classes were lower than the controls (Fig. 1). LN class IV, IV + V and V had significantly higher %PTC than class II (Figs. 1 and 2). The respective percentage of patients with decreased %PTC (<6.02 %), normal %PTC (6.02-9.90 %), and increased %PTC (>9.90 %) was 100, 0,0 in class II LN; 83, 0, 17 in class III LN; 76, 12, 12 in class IV LN, 67, 33, 0 in class IV + V, and 75, 9, 16 in class V LN. All class II LN patients had a lower %PTC area than the control group and morphologically the PTC were without dilatation or abnormal features. In these subjects the %PTC area positively correlated with eGFR. The PTC of class IV or V LN patients who had normal or high %PTC area, however, showed a distinctly abnormal morphology; with the PTC being dilated and some showing infiltrating mononuclear cells within and around the PTC—a characteristic of peritubular capillaritis (Figs. 3 and 4). The class IV LN patients with normal to increased %PTC also showed relatively worse renal function than the reduced %PTC group (i.e., proportion of severe decreased GFR, < 15 mL/min/1.73 m^2^, 14.3 % vs. 6.5 %, *p* = 0.002). By comparison, the class V LN patients with normal to increased %PTC showed a higher degree of hematuria than the decreased %PTC participants (7.4 ± 8.2 vs. 1.7 ± 3.1 cells/hpf, *p* = 0.002).

The following clinical factors were evaluated in relation to PTC loss by univariate analysis: age, sex, blood pressure, eGFR, proteinuria, hematuria, pyuria, presence of crescent, level of complements, and severity of tubulointerstitial damage. Although some clinical factors were significantly associated with % PTC changes, the increase or decrease of PTC did not consistently correlate with the severity of these factors. For example, the % PTC area was increased in patients with modest microscopic hematuria, e.g., % PTC in the mild hematuria group (urine RBC 3–10 cells/hpf, %PTC 5.78 ± 5.17) was higher than in the no hematuria group (mean difference 2.22, 95 % CI 0.69 to 3.75, *p =* 0.005), but in the severe microscopic hematuria groups (i.e., urine RBC > 30 cells/hpf, %PTC 3.21 ± 3.91), %PTC was significantly decreased compared with the mild hematuria group (mean difference −2.57, 95 % CI −4.33 to −0.82, *p =* 0.004).

In addition, the %PTC were lower in the eGFR 15–59 mL/min/1.73 m^2^ group compared to the eGFR ≥ 60 mL/min/1.73 m^2^ group (mean difference 1.13, 95 % CI −2.26 to −0.01, *p =* 0.037). The %PTC was not, however, further decreased in the severe decreased GFR (<15 mL/min/1.73 m^2^) group.

**Fig. 5:**
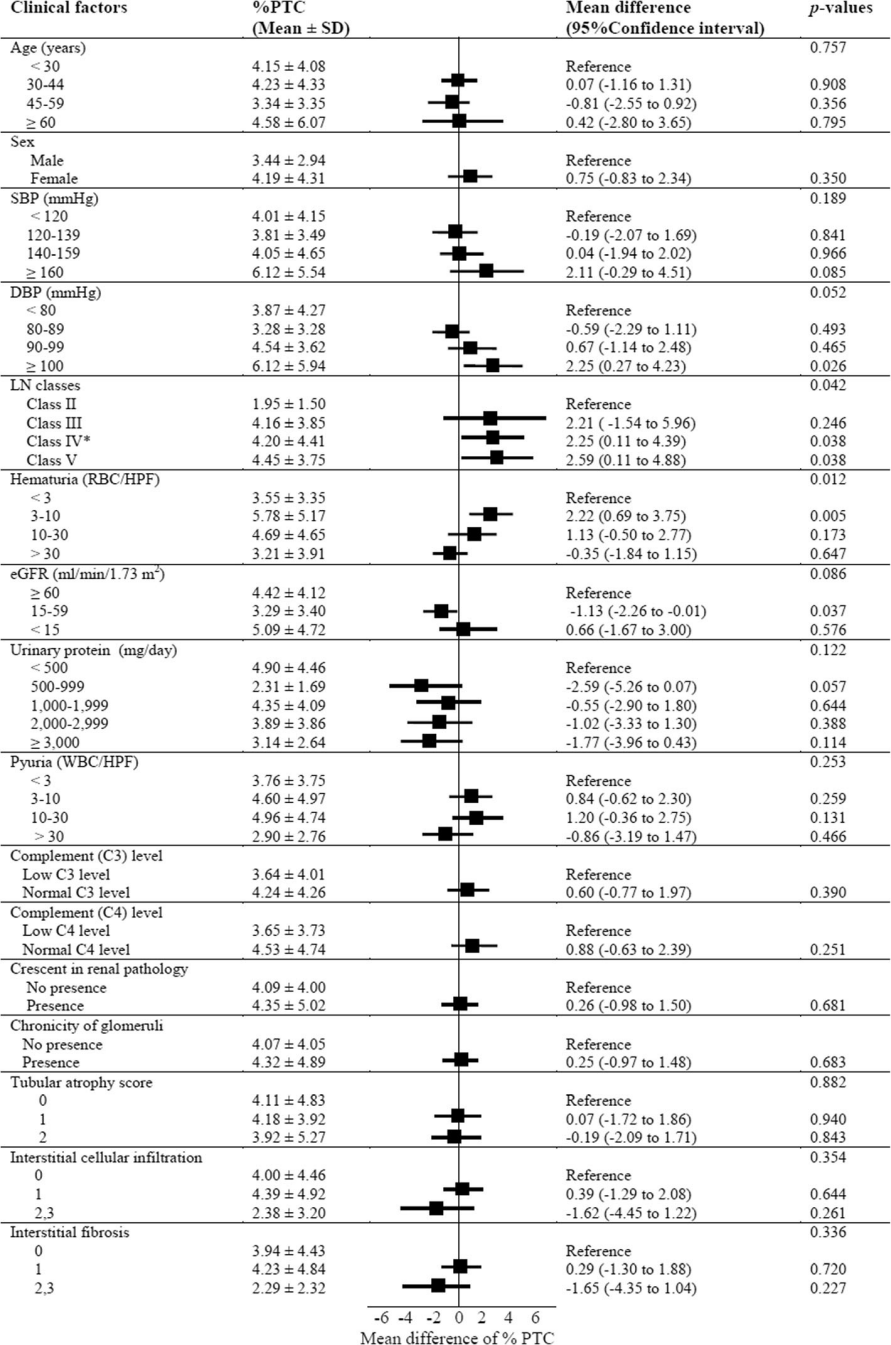
Univariate analysis of the various clinical factors associated with %PTC Notes: RBC; red blood cells, HPF; high power field, eGFR (estimated glomerular filtration rate) defined according to CKD-EPI formula, *included LN class IV + V

Finally, the %PTC area trended to be lower in the presence of proteinuria, however, the change of %PTC also depended on severity of proteinuria. The %PTC in the mild proteinuria group (500–999 mg/day) appeared lower than in the no proteinuria group, although this did not reach statistical significance (mean difference −2.59, 95 % CI −5.26 to 0.07, *p =* 0.057). The %PTC also trended to decline in the severe proteinuria group (≥3,000 mg/d) vs. the moderate proteinuria group (1000–1,999 mg/d) with a mean difference of −1.22, 95 % CI −0.13 to 2.56, *p* = 0.076. In addition, the %PTC was higher in subjects with elevated diastolic blood pressure (DBP ≥ 100 mmHg) group compared to the group with DBP < 80 mmHg (mean difference 2.25, 95 % CI 0.27 to 4.23, *p =* 0.026). Data are summarized in Fig. 5.

The results of multivariate linear regression analysis identified LN classes, microscopic hematuria and renal function to be significantly associated with PTC changes (Table 2). When comparing the LN class II with class III, IV and V, a statistically significant increase in %PTC was found in classes IV (mean difference 2.43, 95 % CI 0.02 to 4.84, *p* < 0.05) and V (mean difference 2.46, 95 % CI 0.02 to 5.00; *p* < 0.05) —likely due to the presence of abnormal dilated vessels (angiogenesis) associated with inflammation.

**Table 2:** Multivariate analysis of the clinical factors associated with percent of PTC change

Clinical factors	%PTC (Mean ± SD)	Unadjusted mean difference (95 % Confidence interval)	Adjusted mean difference (95 % Confidence interval)	*p*-value
LN classes				
II	1.95 ± 1.50	Reference	Reference	
III	4.16 ± 3.85	2.21 (−1.54 to 5.96)	2.67 (−1.79 to 7.13)	0.239
IV^a^	4.20 ± 4.41	2.25 (0.11 to 4.39)	2.43 (0.02 to 4.84)	0.042**
V	4.45 ± 3.75	2.59 (0.11 to 4.88)	2.46 (0.02 to 5.00)	0.047**
Hematuria (cells/HPF)				
<3	3.55 ± 3.35	Reference	Reference	
3–10	5.78 ± 5.17	2.22 (0.69 to 3.75)	1.96 (0.25 to 3.67)	0.025**
10–30	4.69 ± 4.65	1.13 (−0.50 to 2.77)	0.92 (−1.03 to 2.88)	0.353
>30	3.21 ± 3.91	−0.35 (−1.84 to 1.15)	−0.11 (−1.92 to 1.70)	0.904
eGFR (mL/min/1.73 m^2^)				
≥60	4.42 ± 4.12	Reference	Reference	
15–59	3.29 ± 3.40	−1.13 (−2.26 to −0.01)	−1.32 (−2.62 to-0.02)	0.046**
<15	5.09 ± 4.72	0.66 (−1.67 to 3.00)	−0.18 (−2.70 to 2.34)	0.117
Urinary protein (mg/day)				
<500	4.90 ± 4.46	Reference	Reference	
500–999	2.31 ± 1.69	−2.59 (−5.26 to 0.07)	−2.45 (−5.17 to 0.27)	0.077
1,000–1,999	4.35 ± 4.09	−0.55 (−2.90 to 1.80)	−1.04 (−3.47 to 1.39)	0.399
2,000–2,999	3.89 ± 3.86	−1.02 (−3.33 to 1.30)	−1.36 (−3.79 to 1.07)	0.269
≥3,000	3.14 ± 2.64	−1.77 (−3.96 to 0.43)	−2.08 (−4.31 to 0.15)	0.067

Notes: %PTC; percentage of peritubular capillary, eGFR; estimated glomerular filtration rate, calculated according to CKD-EPI formula

^a^included LN class IV + V, **statistical significant *p* < 0.05

The %PTC area in the mild hematuria group was also higher than the no hematuria group (mean difference 1.96, 95 % CI 0.25 to 3.67, *p* < 0.05). The eGFR 15–59 mL/min/1.73 m^2^ group had a %PTC area that was lower than the eGFR > 60 mL/min/1.73 m^2^ group (mean difference −1.32, 95 % CI −2.62 to −0.02, *p* < 0.05). The %PTC trended to be less in those with nephrotic-ranged proteinuria group (mean difference −2.08, 95 % CI −4.31 to 0.15, *p* = 0.067). The reason that no further decrease in %PTC area was observed in the most severe GFR group (i.e., GFR < 15 mL/min/m^2^) may be due to the fact that all of these subjects had class IV LN in which 42 % of them showed normal to high %PTC, while only 12 % and 28 % in the GFR 15–59 and GFR ≥ 60 mL/min/1.73 m^2^ group revealed normal to high %PTC (*p* = 0.005). Furthermore, the %PTC area in the mild hematuria group was highest because it had the greatest proportion of participants who exhibited normal to high %PTC, especially class V LN patients (i.e., 35 % in this group vs. 15–16 % in other degree of hematuria groups, *p* = 0.014). A subgroup analysis of class V LN patients showed 72, 21 and 7 % of them had urine RBC < 3, 3–10 and 10–30 cells/hpf, respectively. Mean %PTC of the urine RBC 3–10 cells/hpf group was significantly higher than those with urine RBC < 3 cells/hpf group (7.33 ± 4.81 vs. 3.51 ± 3.02, *p* = 0.006).

## Discussion

In this study we performed an analysis of over 250 renal biopsies from patients with lupus nephritis in order to test the hypothesis that a reduction in PTC (as noted by PTC density) would be associated with worse renal function. While a prior study had reported a reduction in PTC in LN, the study consisted of 20 biopsies [17] and hence we deemed it important to evaluate a larger sample. Several key findings were noted. First, we confirmed that PTC was reduced in all classes of LN. Second, we found that subjects with stage 3–4 CKD showed more PTC loss than those with CKD stage 2. Finally, we found a tendency for less PTC in subjects with nephrotic range proteinuria. These data generally agree with studies in other glomerular diseases that confirm a relationship with lower PTC and severity of renal disease [14, 21, 24–28].

One of our most important findings was that there was a 40–50 % reduction in PTC in biopsies from LN compared to healthy controls. One aspect of this is that subjects with class II LN showed some of the greatest loss of PTC, which is surprising as they are considered to have relatively mild disease compared to class IV and V LN. However, a review of the biopsies showed that while a loss of PTC is commonly observed in LN, in approximately 25 % of subjects with more severe classes of LN, there was evidence for marked renal inflammation and de novo angiogenesis as noted by the presence of atypical dilated capillaries and capillaritis. This latter finding is typical for neoangiogenesis and has been noted in some models of kidney disease [15, 16]. This is likely due to a period of increased VEGF-A expression during acute inflammation as macrophages are a rich source of cytokine-induced VEGF-A [29, 30]. Although decreased renal VEGF-A expression has been reported in some LN patients as postulated to be a consequence of podocyte injury [17, 31, 32]—some subjects with LN show increased serum and urine VEGF-A levels and increased renal VEGF-A expression [33–37].

Interestingly, the subjects with the increase in PTC due to abnormal angiogenesis did not have preserved renal function, but rather demonstrated worse renal function within their class. Indeed, these subjects showed more severely declined GFR in class IV LN and greater hematuria in class V LN groups. It seems likely that the injury is more severe as a consequence of both greater inflammation [38] while the presence of abnormal vessels and capillaritis may not translate into improved blood flow to the damaged kidney. Consistent with these findings is the report of peritubular capillaritis with tubulointerstitial nephritis in a SLE patient [18].

In our study, subjects with nephrotic-range proteinuria trended to show a greater reduction of PTC. Katavetin et al. proposed that the loss of PTC could be secondary to albuminuria associated reduction in renal tubular VEGF-A production [39]. Nevertheless, we also observed a loss of PTC in subjects with Class II LN that have relatively mild proteinuria. This suggests other mechanisms may be driving PTC loss in these patients. It is of interest that one study reported that a loss of PTC was also high in subjects with mesangial proliferative nephritis compared to other, more severe forms of GN [21]. Again, this may relate to the potential for inflammatory diseases and the extent of renal damage to undergo an initial increase in neovessels with inflammation prior to a loss of PTC as the kidney disease advances [16]. It is possible that milder diseases bypass this initial inflammatory expansion of capillaries. Additionally, some class II LN patients may be transformed from class III, IV or V LN groups after treatment thus the inflammation has already diminished. To confirm this hypothesis, serial changes in the PTC should be analyzed in association with changes in VEGF-A, inflammation and fibrosis.

Limitations of the study include its cross-sectional nature. Confirmation of CD31 expressed on PTC with the other endothelial markers such as CD34 or Ki67 was not performed due to limited samples and would have been useful as a second marker for endothelial cells or endothelial cell proliferation. A strength of this study is the large number of biopsies studied and the masked quantification of the PTC area by two pathologists (correlation coefficient of 0.96).

## Conclusions

PTC loss is common in LN. Some subjects, particularly with more severe LN, may show evidence for increased angiogenesis in the setting of increased inflammation. Both overall PTC loss, as well as increased neoangiogenesis, are associated with worse renal function. Our study emphasizes the importance of PTC in glomerular disease and in subjects with LN. Further study is needed to understand the mechanisms and mediators regulating angiogenesis in LN patients.

## Additional file

Additional file 1: The inter-rater agreement between the two pathologists by using 40 renal samples. (DOCX 27 kb)

## Abbreviations

CI: Confidence interval
CKD: Chronic kidney disease
DAB: Diaminobenzidine tetrahydrochloride
DBP: Diastolic blood pressure
eGFR: Estimated glomerular filtration rate
GN: Glomerulonephritis
ISN/RPS: The International Society of Nephrology/Renal Pathology Society
LN: Lupus nephritis
PBS: Phosphate buffered saline
PECAM-1: Platelet endothelial cell adhesion molecule 1
PTC: Peritubular capillaries
RBC: Red blood cell
SBP: Systolic blood pressure
SLE: Systemic lupus erythematosus
VEGF-A: Vascular endothelial growth factor-A
WBC: White blood cell
