# Supplementary material for: Relation of peritubular capillary features to class of lupus nephritis
Source: BMC Nephrol. 2016 Nov 9;17:169. doi: 10.1186/s12882-016-0388-2 (PMC5103390; doi:10.1186/s12882-016-0388-2)
Supplement: Additional file 1: — The inter-rater agreement between the two pathologists by using 40 renal samples. (DOCX 27 kb) [file 12882_2016_388_MOESM1_ESM.docx]

**The inter-rater agreement between the two pathologists by using 40 renal samples**

Concordance correlation coefficient (Lin, 1989, 2000):

| rho_c SE(rho_c) Obs [ 95% CI ] P CI type |
| --- |
| --------------------------------------------------------------- |
| 0.963 0.008 40 0.946 0.979 0.000 asymptotic |
| 0.942 0.976 0.000 z-transform |
|  |
| Pearson's r = 0.982 Pr(r = 0) = 0.000 C_b = rho_c/r = 0.981 |
| Reduced major axis: Slope = 1.213 Intercept = -0.195 |
|  |
| Difference = rater1 - rater2 |
|  |
| Difference 95% Limits Of Agreement |
| Average Std Dev. (Bland & Altman, 1986) |
| --------------------------------------------------------------- |
| 0.227 1.280 -2.281 2.736 |
